# Assessing physical workload among people with musculoskeletal disorders: validity and reliability of the physical workload questionnaire

**DOI:** 10.1186/s12891-022-05222-y

**Published:** 2022-03-24

**Authors:** Lise Grethe Kjønø, Rikke Munk Killingmo, Ørjan Nesse Vigdal, Margreth Grotle, Kjersti Storheim

**Affiliations:** 1 Faculty of Health Sciences, Oslo Metropolitan University, Oslo, Norway; 2 Research and Communication Unit for Musculoskeletal Health (FORMI), Division of Clinical Neuroscience, Oslo University Hospital, Oslo, Norway

**Keywords:** Physical workload questionnaire, PWQ, Physical workload, Musculoskeletal disorders, Validity, Reliability

## Abstract

**Background:**

Demands of physical work are related to musculoskeletal disorders, and hence, important to assess. The Physical Workload Questionnaire (PWQ) is based on 26 items related to physical workload. The PWQ has been translated into Norwegian, but its psychometric properties have not yet been tested. The aim of this study was to assess the validity and reliability of the PWQ among patients with musculoskeletal disorders.

**Methods:**

A cross-sectional study with a test-retest design was conducted to assess construct validity (structural validity and hypothesis testing) and reliability (internal consistency and test-retest reliability) among employed patients with musculoskeletal disorders. Exploratory factor analysis was performed to assess the structural validity and number of items to be included in the Norwegian version of the PWQ. Hypothesis testing was assessed by 14 a priori hypotheses (“known” group, convergent and discriminant validity). Internal consistency was evaluated using Cronbach’s alpha and test-retest reliability by Intraclass Correlation Coefficient (ICC_2.1_), Standard Error of Measurement (SEM_agreement_) and Smallest Detectable Change (SDC_95%_ind).

**Results:**

In total, 115 patients with a mean age (SD) of 46 (9) years were included, of which 48 were included in the reliability analyses. Exploratory factor analysis resulted in two subscales: “Heavy physical work” (15 items, range 0–100) and “Long-lasting postures and repetitive movements” (7 items, range 0–100). No floor or ceiling effects were seen in the subscales. Twelve of the 14 (85%) predefined hypotheses were confirmed. The internal consistency with Cronbach’s alpha was 0.94 and 0.85 on subscales 1 and 2, respectively. Test-retest reliability analyses demonstrated an ICC_2.1_ of 0.96 (95% CI 0.88, 0.98) and 0.92 (95% CI 0.81, 0.96), SEM of 6.9 and 10.0 and SDC_95%_ind of 19.2 and 27.7 of subscales 1 and 2, respectively.

**Conclusions:**

The Norwegian version of the PWQ demonstrated good validity and reliability and can be used to evaluate physical workload in patients with musculoskeletal disorders.

**Supplementary Information:**

The online version contains supplementary material available at 10.1186/s12891-022-05222-y.

## Background

The demands of physical work are associated with the development of various musculoskeletal disorders [1, 2], and to labour market participation [3, 4]. Work-related musculoskeletal disorders are the leading cause of sickness absence in Europe [5, 6]. According to existing literature, major risk factors for work-related musculoskeletal disorders are heavy lifting, working with a bent or twisted back or elevated arms, repetitive movements, and vibration [1, 2].

To prevent and reduce work-related musculoskeletal disorders, it is necessary to assess physical workload at the workplace [7]. The Physical Workload Questionnaire (PWQ) was developed by Bot et al. [8], with the aim of creating a short and simple self-report questionnaire for assessing physical workload in occupational health care and epidemiological research. Twenty-six items that were expected to have an association with either upper or lower extremity complaints were tested for dimensionality, internal consistency, and construct validity in a population with upper- and lower extremity musculoskeletal disorders in the Netherlands. The items formed two subscales and the results supported the internal structure, internal consistency, and construct validity [8], suggesting that PWQ is useful for assessing physical workload in a population with musculoskeletal disorders. To the best of our knowledge, the PWQ has not been translated into any other languages or been tested for its psychometric properties in other studies, hence there is a need for assessment of the questionnaire in a different population and among patients with a broader range of musculoskeletal disorders. The PWQ was previously translated and cross-culturally adapted into Norwegian according to international guidelines [9, 10]. The present study aims to test the PWQʼs validity and reliability in terms of structural validity, hypothesis testing, internal consistency, and test-retest reliability among Norwegian patients with various musculoskeletal disorders.

## Methods

This study was designed and performed in accordance with the COSMIN checklist [11] and guidelines for PROMs [12].

### Design

We used a cross-sectional design, including a test-retest assessment.

### Translation and cross-cultural adaptation

The translation and cross-cultural adaptation were done according to international guidelines [9, 10]. Two translators (one philologist and one clinician), whose mother tongue is Norwegian, independently translated the 26 items into Norwegian and synthesized them into one Norwegian version before it was translated back to English. Two translators and native English speakers, blinded the original PWQ items, independently performed the backtranslation and synthesized the two versions into one English version. An expert committee consisting of the translators and two researchers from the research group (MG, RMK) reviewed the translations and agreed on a prefinal version. Ten patients with musculoskeletal disorders reviewed the prefinal Norwegian version. The items and responses were confirmed to be relevant and understandable without any proposed alterations. Since the prefinal version was acceptable and easy to comprehend, no changes were made for the final version.

### Participants

Participants were recruited from an outpatient rehabilitation clinic in Akershus, Norway, between November 2015 and January 2018. Eligible participants were patients with different types of musculoskeletal disorders, aged 18 or above, working or on sick leave, who were referred to a specialist assessment and rehabilitation at the outpatient rehabilitation clinic. Exclusion criteria were patients being unable to speak, read or write in Norwegian. Inclusion was performed by clinicians, primarily physiotherapists, meeting with patients at the clinic. At baseline, all patients received written and oral information about the study, and provided their signed, informed consent.

According to recommended quality criteria by Terwee et al. [12] and Kline [13] we planned to recruit a minimum of 100 patients. These criteria suggest a minimum of 100 participants for assessing internal consistency, at least 50 participants for assessing reliability and floor or ceiling effects [12], and at least 4–10 participants for each item included in factor analysis [13].

### Procedures and measurements

At baseline, patients completed the PWQ as part of a comprehensive questionnaire which also included sociodemographic variables, pain localization, intensity and history, psychosocial work environment, productivity costs and health-related quality of life.

The McGill pain drawing was used to measure pain localisation during the last week [14]. The Numeric Rating Scale (NRS) (range 0–10, a higher score indicates more severe pain) was used to measure average pain intensity in the last week [15]. The General Nordic Questionnaire for psychological and social factors at work (QPSnordic) was used to measure characteristics of the psychosocial work environment [16]. The iMTA Productivity Cost Questionnaire (iPCQ) was used to measure work status (occupation, paid job, working days/hours a week, sick leave and rehabilitation/work disability) and productivity costs [17]. The Short Form 36 Health Status Questionnaire (SF-36) (range 0–100, higher score indicates better health- related quality of life) was used to measure health-related quality of life. In addition, the Mechanical Exposure Index (MEI) (range 0–24, higher score indicates higher physical workload) was used to measure physical workload [18].

Patients consenting to participate in the test-retest part of the study filled out the PWQ and a global question recording change in work status at a second meeting, preferably within 1 week. Patients reporting “unchanged” work status were considered stable and included in the test-retest reliability analysis.

### The physical workload questionnaire

The PWQ is a self-report questionnaire for assessing physical workload [8]. The questionnaire consists of 26 items assessing force, dynamic and static load, repetitive load, (uncomfortable) postures, sitting, standing, and walking. In the only previous study, assessing dimensionality, internal consistency, and construct validity among patients with upper and lower extremity musculoskeletal disorders in the Netherlands, factor analysis revealed two subscales- twelve items related to the first subscale “Heavy physical work” and six items related to the second subscale “Long-lasting postures and repetitive movements” [8]. The remaining eight items were excluded due to low loading or to similar loading on both subscales. Each item is scored on a 4-point Likert scale with the response options: “seldom or never” (0), “sometimes” (1), “often” (2), and “(almost) always” (3). Scoring is conducted by adding up the responses to each item to produce a raw score. The final scores are calculated by dividing the raw score by the maximum possible score on the subscale, multiplied by 100, resulting in a final score ranging between 0 (no workload) and 100 (highest workload) for each subscale [8]. The Norwegian version of the 26 items on the PWQ is shown in Additional file 1.

### Analysis

All data analyses were performed using SPSS version 26 (IBM Corporation, Armonk, NY, USA). The structural validity was explored using Exploratory Factor Analysis (EFA) based on the same 26 items which formed the basis of the study of Bot et al. [8]. The suitability of data for factor analysis was confirmed using the Kaiser-Meyer-Olkin measure of sampling adequacy (values above 0.6 considered acceptable), a significant Bartlettʼs Test of Sphericity and inspection of the correlation matrix (correlation coefficients of .3 and above preferable) [19]. Principal Component Analysis (PCA) was used to extract the factors followed by oblique rotation of factors using oblimin rotation. The number of factors to be retained was guided by three decision rules: Kaiserʼs criterion, retention of eigenvalues above 1, Cattelʼs scree plot [20], and by the use of Hornʼs parallel analysis [21]. To aid in the interpretation of the retained factors, we computed factor loadings after direct oblimin rotation, allowing factors to correlate [19]. The next step involved interpreting the rotated solution by identifying which items loaded on each retained factor. Items with factor loading below 0.5 [22] and communalities value below 0.3 were excluded [23]. Items which cross-loaded were retained in the factor they loaded most strongly.

Hypothesis testing was assessed by 14 a priori hypotheses; “known” group validity (eight), convergent validity (two) and discriminant validity (four). The “known” group hypothesis are identical to those in the original study. They were tested with the same procedure as in the study of Bot et al., where it was hypothesised that physical workload would vary among different occupational groups [8]. As in the original study, the occupations of all included patients were classified into four groups based on expected physical load, and the subscale scores of the occupational groups were compared.

- Group 1: no physical load (for example teacher, manager)
- Group 2: heavy physical load (for example nurse, childcare worker)
- Group 3: long-lasting postures and repetitive movements (for example cashier, civil servant, engineer)
- Group 4: both heavy physical load and long-lasting postures and repetitive movements (for example electrician, farmer, mechanic)

Two investigators (LGK, ØNV) made the classifications independently, based on available occupation descriptions [24, 25]. Disagreements were resolved in a consensus meeting with a third investigator (RMK). Three occupations could not be classified (police, shop assistant and service employee) due to considerable physical workload variability within the occupations, and patients with these occupations were therefore excluded from the hypothesis analyses.

To assess convergent validity, both subscales were validated against the MEI [18]. The MEI includes similar questions to the PWQ, especially regarding heavy physical workload. We therefore expected high correlation between the MEI and the “Heavy physical work” subscale and moderate to high correlation between the MEI and the “Long-lasting postures and repetitive movements” subscale. To assess discriminant validity of the PWQ subscales, we formulated hypotheses regarding two dimensions from SF-36; “physical function” and “general health” [26]. These dimensions measure different constructs to the PWQ. We therefore expected low correlation between both PWQ subscales and the SF-36 dimensions. If > 75% of the predefined hypotheses were confirmed, construct validity was considered acceptable [12]. Mann-Whitney U tests and Wilcoxon signed ranks tests were used in “known” group analyses. Spearman’s rho was used in all correlation analyses (convergent and discriminant validity) because the scales were not normally distributed. Correlation coefficients under 0.3, between 0.3 and 0.6 and over 0.6 were considered low, moderate and high, respectively [27]. The hypotheses are listed in Table 3.

The internal consistency of the subscales was examined using Cronbach’s alpha. Cronbach’s alpha between 0.70 and 0.95 gave a positive rating [12]. The item-total correlation was examined and items with values below 0.3 were excluded [28].

For test-retest assessment, a paired t-test was used to assess the mean difference between test and re-test. An intraclass correlation coefficient (ICC_2,1_) was used to assess relative reliability. The acceptable level of ICC was set to ≥0.70 [12]. Absolute reliability (measurement error) was evaluated by standard error of measurement (SEM) and smallest detectable change (SDC). ICC_2.1_ and SEM_agreement_ were used to account for the systematic difference between test and re-test [28]. SEM was estimated from the SPSS VARCOMP analysis; SEM_agreement_ =√ (o^2^_o_ + o^2^_po,e_), where o^2^_o_ is the variance due to systematic error between observations and o^2^_po,e_ is the random error. Based on this, the SDC was estimated using the formula SDC_95%_ind = 1.96 × √2 x SEM_agreement_ [28].

Proportions of missing data and floor and/or ceiling effects were described. Floor or ceiling effects were considered to be present if more than 15% of patients reported either the lowest or the highest possible score [12].

## Results

A total of 115 patients with a mean (SD) age of 46 (9) were included. Study sample characteristics are presented in Table 1. The majority of the patients were women. Almost all patients (90%) were in paid work, and more than half had been on sick leave during the previous 4 weeks. On average, patients reported moderate pain severity, the majority had pain for more than 3 months, and the most frequently reported pain area was the back region. Physical workload was generally low.

**Table 1:** Patient demographic characteristics and clinical status at baseline

	Cross sectional study sample		Test-retest study sample	
	***n*** =		***n*** =	
Female, N (%)	115	79 (68.7)	48	30 (62.5)
Age in years, mean (SD)	115	45.6 (9.3)	48	46.2 (8.6)
Mother tongue Norwegian, N (%)	115	100 (87)	48	43 (89.6)
Educational level high, N (%)	115	67 (58.2)	48	30 (62.5)
Work status, N (%)				
Employed or self-employed (paid job)	115	104 (90.4)	48	42 (87.5)
Working hours per week, median (range)	111	37.5 (7.5–52)	47	37.5 (7.5–52)
Working days per week, median (range)	102	5 (2–7)	41	5 (2–6)
Sick leave during last 4 weeks	114	79 (68.7)	48	33 (68.8)
Rehabilitation, work disability	115	7 (6.1)	48	4 (8.4)
Pain location, N (%)				
Lower extremities		70 (60.9)		27 (56.3)
Back		80 (69.6)		36 (75.0)
Neck		57 (49.6)		17 (35.4)
Upper extremities		55 (47.8)		15 (31.3)
Pain duration in months, median (range)	89	8.3 (0.2–360)	38	8.6 (0.2–360)
≤ 3 months, N (%)		13 (14.6)		5 (13.2)
> 3 months, N (%)		76 (85.4)		33 (86.8)
Pain severity in the last week (NRS 0–10), mean (SD)	112	5.2 (2.0)	48	4.9 (2.2)
Physical Workload Questionnaire (PWQ 0–100) Heavy physical work, median (range)	107	15.6 (0–82.2)	48	16.7 (2.2–82.2)
Long-lasting postures and repetitive movements, mean (SD)	110	49.1 (25.6)	48	49.2 (25.4)
Mechanical exposure index (MEI 0–24), median (range)	111	4 (0–20)	47	4 (0–20)
Health-related quality of life (SF-36 0–100), median (range)				
Physical function	115	75 (30–100)	48	70 (30–95)
General health	115	57 (5–97)	48	68.5 (15–97)

*NRS* Numeric rating scale (0 = no pain, 10 = worst possible pain), *PWQ* Physical Workload Questionnaire (0 = no workload, 100 = highest workload), *MEI* Mechanical Exposure Index (0 = no workload, 24 = highest workload), SF-36: The short form 36 Health status questionnaire (0 = maximum disability, 100 = no disability)

Sixty-two patients completed the retest questionnaire, of which 48 reported no change in working conditions and had complete PWQ scores and could thus be included in the test-retest analysis. Patients participating in the test (*n* = 115) and the retest (*n* = 48) were largely similar, however, individuals included in the retest had slightly different pain site locations, physical function, and general health on the SF-36. The median (range) time interval between test and retest was 3 days.

### Structural validity

Inspection of the correlation matrix revealed the presence of many coefficients above 0.3. Bartlettʼs Test of Sphericity was highly significant (*p* < 0.001), and the Kaiser-Meyer-Olkin measure of sampling adequacy value of 0.86 supported the factorability of the correlation matrix [19]. PCA revealed the presence of five factors with eigenvalues exceeding 1 (Kaiserʼs criterion), explaining 39, 16, 6, 4 and 4% of the variance, respectively. However, the results of Hornʼs parallel analysis indicated only two factors appropriate for retention and the scree plot suggested either a three- or two-factor solution, therefore, both the three- and two-factor solutions were inspected.

The three-factor solution explained a total of 61% (39, 16 and 6%) of the variance. Examination of the factor loadings revealed only two items in factor 3 (“sitting for long periods of time” and “visual display units (VDU) work for long periods of time”, and, as a subscale in a questionnaire should be comprised of least three items [28], the three-factor solution was rejected. Therefore, PCA with oblimin rotation was repeated, forcing two factors. The items “sitting for long periods of time” (2) and “VDU work for long periods of time” (3) loaded highly negative on the first factor and below 0.5 on the second factor and were therefore excluded. Item 22 “climbing stairs” was excluded due to negative loading on factor 2 and loading below 0.5 on factor 1. Item 21 “operating peddles with your feet” showed low communalities value (0.276), indicating a poor fit with the other items in the factor, and was therefore excluded. Results from the three- and two-factor solutions are presented in Additional files 2 and 3.

Finally, a forced two-factor analysis with oblimin rotation on the remaining items was found to explain 58% (41 and 17%) of the total variance (Table 2). The items that loaded highly on the first factor were related to heavy physical work and the items that loaded highly on the second factor were related to static postures or repetitive movements. As a result, 22 items remained (15 items in factor 1 and 7 items in factor 2). The factor labels proposed by Bot et al. [8] suited the extracted factors in this analysis which resulted in: subscale 1 “Heavy physical work” and subscale 2 “Long-lasting postures and repetitive movements”.

**Table 2:** Final factor loadings after forced two factor solution and exclusion of items

Item	Pattern coefficients		Structure coefficients	
Does your work involve …	Factor 1	Factor 2	Factor 1	Factor 2
17. Physical hard work?	**0.84**		**0.86**	
13. Moving loads (more than 5 kg)?	**0.83**		**0.83**	
12. Work (ing) with your hands below knee level?	**0.80**		**0.79**	
11. Work (ing) with your hands above shoulder level?	**0.78**		**0.80**	
23. Squatting often?	**0.78**		**0.74**	
14. Moving heavy loads (more than 25 kg)?	**0.77**		**0.77**	
15. Exerting force with your arms or hands?	**0.75**	0.30	**0.80**	0.42
5. Kneeling or squatting for long periods of time?	**0.75**		**0.75**	
4. Walking long periods of time?	**0.73**		**0.70**	
25. Sitting or moving on your knees?	**0.71**		**0.74**	
16. Exerting maximal force?	**0.70**		**0.74**	
1. Standing for long periods of time?	**0.66**		**0.64**	
24. Walking on irregular surfaces?	**0.61**		**0.64**	
7. Working in a twisted posture for long periods of time?	**0.60**	0.46	**0.61**	**0.56**
20. Working with vibrating tools?	**0.57**		**0.58**	
6. Making the same movement for long periods of time for long periods of time?		**0.83**		**0.82**
18. Working in the same position for long periods of time?	−0.37	**0.82**		**0.76**
10. Holding your wrist in a bent or twisted position for long periods of time?		**0.75**		**0.77**
26. Doing repetitive tasks with arms, hands or fingers many times per minute?		**0.72**		**0.69**
8. Holding your neck in a bent forward or twisted position for long periods of time?		**0.63**		**0.66**
9. Bending or twisting your neck often?	0.33	**0.62**	0.43	**0.67**
19. Working in uncomfortable postures?	0.39	**0.56**	0.49	**0.62**
Eigenvalue	9.15	3.66		
Variance explained	41.6%	16.7%		
Total variance explained	58.3%			

Factor loadings < 0.3 are removed. Factor loadings > 0.5 are given in bold

### Construct validity

In total, 12 (85%) of the 14 predefined hypotheses were confirmed (Table 3), indicating acceptable construct validity.

**Table 3:** Construct validity: A priori formulated hypotheses (*n* = 115)

	Value	Hypothesis confirmed
The mean score on the subscale “heavy physical work” is significantly higher for the occupational group with heavy physical load (*n* = 10) than for the occupational group with long-lasting postures and repetitive movements (*n* = 52)	*p* = 0.008	Yes
The mean score on the subscale “heavy physical work” is significantly higher for the occupational group with heavy physical load (*n* = 10) than for the occupational group without physical load (*n* = 20)	*p* = 0.038	No
The mean score on the subscale “long-lasting postures and repetitive movements” is significantly higher for the occupational group with long-lasting postures and repetitive movements (*n* = 53) than for the occupational group with heavy physical load (*n* = 10)	*p* = 0.001	Yes
The mean score on the subscale “long-lasting postures and repetitive movements” is significantly higher for the occupational group with long-lasting postures and repetitive movements (*n* = 53) than for the occupational group without physical load (*n* = 22)	*p* < 0.001	Yes
The mean score on the subscale “heavy physical work” is significantly higher for the occupational group with both heavy physical load and long-lasting postures and repetitive movements (*n* = 14) than for the occupational group without physical load (*n* = 20)	*p* < 0.001	Yes
The mean score on the subscale “long-lasting postures and repetitive movements” is significantly higher for the occupational group with both heavy physical load and long-lasting postures and repetitive movements (*n* = 13) than for the occupational group without physical load (*n* = 22)	*p* = 0.001	Yes
In the occupational group with heavy physical load (*n* = 10), the mean score on the subscale “heavy physical work” is significantly higher than on the subscale “long-lasting postures and repetitive movements”	*p* = 0.173	No
In the occupational group with long-lasting postures and repetitive movements (*n* = 52), the mean score on the subscale “long-lasting postures and repetitive movements” is significantly higher than on the subscale “heavy physical work”	*p* < 0.001	Yes
High correlation between “heavy physical work” score and the MEI score (*n* = 104)	rho = 0.783 *p* < 0.001	Yes
Moderate to high correlation between “long-lasting postures and repetitive movements” score and the MEI score (*n* = 106)	rho = 0.402 *p* < 0.001	Yes
Low correlation between “heavy physical work” score and “physical function” score (SF-36) (*n* = 107)	rho = -0.288 *p =* 0.003	Yes
Low correlation between “long-lasting postures and repetitive movements” score and “physical function” score (SF-36) (*n* = 110)	rho = -0.155 *p =* 0 .107	Yes
Low correlation between “heavy physical work” score and “general health” score (SF-36) (*n* = 107)	rho = -0.126 *p* = 0.195	Yes
Low correlation between “long-lasting postures and repetitive movements” score and “general health” score (SF-36) (*n* = 110)	rho = -0.282 *p =* 0.003	Yes

Hypotheses were analysed with Mann-Whitney U tests, Wilcoxon signed ranks tests (both presented with *p* values) and correlation analysis (presented with Spearman’s rho and *p* values)

*MEI* Mechanical Exposure Index, *SF-36* Short Form 36 Health Status Questionnaire

### Internal consistency

The Cronbach alpha value was 0.94 and 0.85 for subscale 1 and 2, respectively. The item-total correlation was 0.53–0.84 and 0.52–0.73 for subscale 1 and 2 respectively, indicating that all items correlated well with the total subscales.

### Test-retest reliability

Relative and absolute reliability values for patients reporting no change in physical workload are presented in Table 4. Both subscales showed acceptable relative reliability (ICC_2,1_ > 0.7).

**Table 4:** Test-retest reliability

	First test	Re-test	Difference	ICC_2.1_ (95% CI)	SEM_agreement_	SDC_95%_ ind
Heavy physical work *(n = 48)*	27.3 (24.7)	22.2 (21.9)	−5.1 (8.5)*	0.96 (0.88, 0.98)	6.9	19.2
Long-lasting postures and repetitive movements *(n = 48)*	49.2 (25.4)	42.7 (24.1)	−6.5 (12.6)*	0.92 (0.81, 0.96)	10.0	27.7

*ICC* Intraclass correlation coefficient, *SEM* Standard error of measurement, *SDC* Smallest detectable change

Score values and difference values are presented with mean (SD)

*P values < 0.01

The proportion of missing data was very small, under 4% for all single items (ranging from 0.9 to 3.5%). For subscales 1 and 2, missing data was 7% and 4,3%, respectively. There were no floor or ceiling effects for any of the subscales. However, there were ceiling effects on five single items and floor effects on all but two single items. The highest floor effect was 79,8% (Additional file 4).

## Discussion

In this study, the validity and reliability of the PWQ were found to be good when assessed in a sample with various musculoskeletal disorders.

Since the population in our study was made up of patients with various musculoskeletal disorders, and therefore different from the population in the original study (mainly upper- and lower extremity musculoskeletal disorders) [8], all 26 questions were included in the factor analyses. Factor analysis revealed that the PWQ could be divided into two subscales with a total of 22 items remaining in the final PWQ when tested in a sample with various musculoskeletal disorders. The present results were in line with those of the original study in terms of the number of subscales obtained and the nature of the items comprising each of the subscales. However, the number of items included in each subscale differed. In the current study, 3 additional items were included in subscale 1 (“working with vibrating tools”, “walking on irregular surfaces” and “working in a twisted posture for long periods of time”), and one more item (“working in uncomfortable postures”) was included in subscale 2. Considering that the current study also included patients with back pain, and that the most frequently reported pain area was the back region, this difference was not entirely unexpected. Back pain is in several previous studies found to be associated with risk factors such as twisted posture [29–31], working with vibrating tools [32] and uncomfortable postures/ awkward postures [29, 30, 33]. This might explain why these items loaded strongly enough to be included in the subscales in the present study. As the PWQ originally was composed for patients with either upper- or lower extremity disorders some items may also not be applicable for those with back pain. For example, the items “neck bent forward”, “turning/bending neck” and “repetitive tasks arms/hands” are items more often associated with neck- and upper limb pain [1, 2].

Regarding “known” group validity, we found that the median values were statistically significantly different between occupational groups for six of the eight hypotheses. In line with Bot et al. [8] we found that the PWQ clearly distinguished between the subscale scores of the occupational group with “long-lasting postures and repetitive movements” as this group scored low on the first subscale and high on subscale two. In addition, all hypotheses regarding occupations classified as “both physical heavy load and long-lasting postures and repetitive movements” were confirmed. In the “known” group analyses, a significance level of 0.01 was chosen to adjust for multiple testing and give more power to the results thereby. However, when decreasing the level of significance, the probability of wrongly accepting the null hypothesis increases, thus increasing the possibility of type 2 error. The hypotheses regarding convergent validity and discriminant validity were confirmed. The MEI, which is a questionnaire assessing mechanical exposure of the shoulder-neck region [18], showed high correlation with the “heavy physical work” subscale and moderate correlation with the “long-lasting postures and repetitive movements” subscale. The SF-36 dimensions “physical function” and “general health” [26] measure constructs other than physical workload, and, as expected, low correlation was found with both subscales. Eighty-five percent of the predefined hypotheses were confirmed, indicating acceptable construct validity [12].

The good internal consistency of the subscales indicated that the items in the respective subscales correlated well with each other, and thus that they measured the same concept [12]. However, a Cronbach’s alpha value exceeding 0.9 may indicate that some items are redundant [22]. Examination of the item-total statistics showed that three items would decrease the Cronbach’s alpha to 0.93 if they were removed from the scale (“moving loads more than 5kg”, “exerting force with your arms and hands” and “physical hard work”). However, the decrease was minimal, and we considered the items to be important and to contribute to the content validity of the instrument. This result is consistent with the original study [8], which showed a Cronbach’s alpha of 0.92–0.93 on subscale 1 and 0.86–0.87 on subscale 2.

The ICC was well above the minimum standard of both subscales and was therefore considered to be acceptable, which suggests that the PWQ is a reliable measure in our population [28]. There was a statistically significant decrease in difference score from test to re-test in both subscales. However, the decrease may be considered to be low as the scale ranged from 0 to 100. The absolute reliability, presented as measurement error and reported in the actual scale unit, is more clinically useful than the decrease in difference score and relative reliability. The SDC_95%ind_ results indicate that a score of self-reported physical workload at the individual level would have to change by 19.2 and 27.7 on subscales 1 and 2, respectively, to ensure that the change was not a result of measurement error [28]. On a scale from 0 to 100, these values may indicate relatively large measurement error. Further research is needed to evaluate the responsiveness and MIC of the PWQ subscales.

### Limitations

The main limitation of this study is aspects of the sample size. Although the sample size of 115 participants was above the minimum threshold for conducting factor analysis, it was rather low in regard to the number of subjects per variable according to the rules of thumb (4 to 10 subjects per variable) [13]. New guidelines from COSMIN, published after the data collection for this study was finished, require a minimum of 7 participants per item to be considered “very good” in the quality criteria [34]. Hence, the sample size of this study may have influenced the robustness of the factor analysis. Guidelines recommend a minimum of 50 participants in test-retest analyses [11, 12]. Although 62 patients participated in the test-retest study, only 48 could be included in the analyses and there might be some imprecision in our estimates regarding test-retest assessment. In addition, our sample were recruited from a clinic located in a wealthy city close to the capital of Norway, which may imply that this study sample consist of patients with high socioeconomic status. Previous studies showed that low socioeconomic status is associated with higher exposure of physical workload [35, 36], and there is reason to believe that by recruiting participants from a wider geographical area we would have reached a broader population regarding occupational variation. This might influence the degree of representativeness to other populations of people in work with musculoskeletal disorders, in particular those with low socioeconomic status and those who are seeking primary health care. A second potential limitation is that we included patients who were on sick leave, in which could potentially introduce recall bias to exposure estimates. Furthermore, the time interval between measurements may be another potential limitation. Test-retest reliability should be assessed in a stable population with an appropriate time interval between measurements [12]. In the current study the time interval was median 3 days (range 1–10), meaning it was shorter than recommended for many of the patients. There is a potential risk of recall bias if the interval between the test and the re-test is too short. However, we believe that the comprehensive questionnaire with a high number of questions used in the first test most likely reduced recall bias when the same questionnaire was filled out only a few days later. In addition, self-ratings may suffer from misclassification. There are studies showing that workers with musculoskeletal disorders may overestimate the physical load compared to healthy workers [37, 38]. Even when participants are motivated to report the workload accurately, they may have difficulties with recalling and accurately reporting the information [39], or that pain level at the day of answering the questionnaire affects the self-reported level of physical workload [40], which may threaten the validity of the questionnaire. We also have a lack of data on eligible patients who declined to participate.

A strength of this study is that we followed the COSMIN checklist and PROM guidelines in the assessment [11, 12]. The number of items was reduced in a systematic manner by performing EFA according to guidelines. Our study also added convergent and discriminant validity to the construct validity and is the first to assess test-retest reliability of the PWQ.

## Conclusions

The PWQ, consisting of two subscales: “Heavy physical work” and “Long-lasting postures and repetitive movements”, showed good validity and reliability when used among patients with long-lasting musculoskeletal disorders receiving rehabilitation in an outpatient clinic in Norway. This study indicates that the PWQ can be used in clinical and occupational healthcare and for research purposes among patients with musculoskeletal disorders. Further research should be conducted on hypothesis testing and test-retest reliability in other populations and clinical settings. As well, the clinical value of the PWQ in relation to work-related musculoskeletal disorders should be investigated.

## Supplementary Information

**Additional file 1.** The Norwegian version of the PWQ.

**Additional file 2.** Table of Pattern and Structure matrix of three-factor solution after EFA with oblimin rotation.

**Additional file 3.** Table of Pattern and Structure matrix of two-factor solution after EFA with oblimin rotation.

**Additional file 4 **Table of missing data, floor- and ceiling effects of the PWQ subscales and items (*n* = 115).

## Data Availability

The datasets generated and analysed during the current study are not publicly available due to protection of the participants’ anonymity but are available from mgrotle@oslomet.no upon reasonable request.

## Abbreviations

CI: Confidence Interval
COSMIN: COnsensus-based Standards for the selection of health Measurement INstruments
EFA: Exploratory Factor Analysis
ICC: Intraclass Correlation Coefficient
iPCQ: The iMTA Productivity Cost Questionnaire
MEI: Mechanical Exposure Index
MIC: Minimal Important Change
NRS: Numeric Rating Scale
PCA: Principal Component Analysis
PROMs: Patient-Related Outcome-Measures
PWQ: Physical Workload Questionnaire
QPSnordic: General Nordic Questionnaire for Psychological and social factors at work
SD: Standard Deviation
SDC: Smallest Detectable Change
SEM: Standard Error of Measurement
SF-36: Short Form 36 Health Status Questionnaire
VDU: Visual Display Unit
