# Supplementary material for: Assessing physical workload among people with musculoskeletal disorders: validity and reliability of the physical workload questionnaire
Source: BMC Musculoskelet Disord. 2022 Mar 24;23:282. doi: 10.1186/s12891-022-05222-y (PMC8944019; doi:10.1186/s12891-022-05222-y)
Supplement: Supplementary file 1 — Additional file 1. The Norwegian version of the PWQ. [file 12891_2022_5222_MOESM1_ESM.docx]

**Additional file 1.** The Norwegian version of the PWQ

| **Spørreskjema for fysisk arbeidsbelastning** | | | | | |
| --- | --- | --- | --- | --- | --- |
| **Innebærer arbeidet ditt…**  (Does your work involve…) | | **sjelden eller aldri**  (seldom or never) | **av og til**  (sometimes) | **ofte**  (often) | **(nesten) alltid**  ((almost) always) |
| 1. | lange perioder hvor du står?  (Standing for long periods of time?) | 1 | 2 | 3 | 4 |
| 2. | lange perioder hvor du sitter?  (Sitting for long periods of time?) | 1 | 2 | 3 | 4 |
| 3. | lange perioder med skjermarbeid?  (VDU work for long periods of time?) | 1 | 2 | 3 | 4 |
| 4. | lange perioder hvor du går?  (Walking long periods of time?) | 1 | 2 | 3 | 4 |
| 5. | lange perioder hvor du står på kne eller på huk?  (Kneeling or squatting for long periods of time?) | 1 | 2 | 3 | 4 |
| 6. | lange perioder hvor du gjentar de samme bevegelsene?  (Making the same movement for long periods of time?) | 1 | 2 | 3 | 4 |
| 7. | lange perioder hvor du arbeider i en vridd stilling?  (Working in a twisted posture for long periods of time?) | 1 | 2 | 3 | 4 |
| 8. | lange perioder hvor du er i en framoverbøyd eller vridd stilling med nakken?  (Holding your neck in a bent forward or twisted position for long periods of time?) | 1 | 2 | 3 | 4 |
| 9. | at du ofte må bøye eller vri hodet?  (Bending or twisting your neck often?) | 1 | 2 | 3 | 4 |
| 10. | lange perioder hvor du har en bøyd eller vridd stilling i håndleddet?  (Holding your wrist in a bent or twisted position for long periods of time?) | 1 | 2 | 3 | 4 |
| 11. | at du må arbeide med hendene over skulderhøyde?  (Work(ing) with your hands above shoulder level?) | 1 | 2 | 3 | 4 |
| 12. | at du må arbeide med hendene under knehøyde?  (Work(ing) with your hands below knee level?) | 1 | 2 | 3 | 4 |
| 13. | at du må forflytte objekter over 5 kg?  (Moving loads (more than 5 kg)?) | 1 | 2 | 3 | 4 |
| 14. | at du må forflytte tunge objekter over 25 kg?  (Moving heavy loads (more than 25 kg)?) | 1 | 2 | 3 | 4 |
| 15. | at du bruker krefter i armer eller hender?  (Exerting force with your arms or hands?) | 1 | 2 | 3 | 4 |
| 16. | at du tar i alt du kan?  (Exerting maximal force?) | 1 | 2 | 3 | 4 |
| 17. | tungt fysisk arbeid?  (Physical hard work?) | 1 | 2 | 3 | 4 |
| 18. | at du arbeider i den samme stillingen i lange perioder?  (Working in the same position for long periods of time?) | 1 | 2 | 3 | 4 |
| 19. | at du arbeider i ukomfortable stillinger?  (Working in uncomfortable postures?) | 1 | 2 | 3 | 4 |
| 20. | arbeid med vibrerende verktøy/instrumenter?  (Working with vibrating tools?) | 1 | 2 | 3 | 4 |
| 21. | at du bruker eller styrer pedaler med føttene?  (Operating peddles with your feet?) | 1 | 2 | 3 | 4 |
| 22. | at du går i trapper?  (Climbing stairs?) | 1 | 2 | 3 | 4 |
| 23. | at du ofte går opp og ned på huk?  (Squatting often?) | 1 | 2 | 3 | 4 |
| 24. | at du går på ujevnt underlag?  (Walking on irregular surfaces?) | 1 | 2 | 3 | 4 |
| 25. | at du sitter eller beveger deg på knærne?  (Sitting or moving on your knees?) | 1 | 2 | 3 | 4 |
| 26. | repeterende arbeidsoppgaver med armene, hendene eller fingrene mange ganger i minuttet?  (Doing repetitive tasks with arms, hands or fingers many times per minute?) | 1 | 2 | 3 | 4 |

Physical workload questionnaire etter Bot SD et al. [Occup Environ Med.](http://www.ncbi.nlm.nih.gov/pubmed/15550603) 2004 Dec;61(12):980-6: Internal consistency and validity of a new physical workload questionnaire. Oversatt av Grotle M og Munk R 2014, HiOA.
