# Supplementary material for: Assessing physical workload among people with musculoskeletal disorders: validity and reliability of the physical workload questionnaire
Source: BMC Musculoskelet Disord. 2022 Mar 24;23:282. doi: 10.1186/s12891-022-05222-y (PMC8944019; doi:10.1186/s12891-022-05222-y)
Supplement: Supplementary file 2 — Additional file 2. Table of Pattern and Structure matrix of three-factor solution after EFA with oblimin rotation. [file 12891_2022_5222_MOESM2_ESM.docx]

**Additional file 2.** Table of Pattern and Structure matrix of three-factor solution after EFA with oblimin rotation

| **Item** | **Pattern coefficients** | |  | **Structure coefficients** | | |
| --- | --- | --- | --- | --- | --- | --- |
| Does your work involve… | **Factor 1** | **Factor 2** | **Factor 3** | **Factor 1** | **Factor 2** | **Factor 3** |
| 24. Walking on irregular surfaces? | **0.84** |  |  | **0.78** |  |  |
| 25. Sitting or moving on your knees? | **0.76** |  |  | **0.79** |  | -0.39 |
| 22. Climbing stairs? | **0.74** |  |  | **0.59** |  |  |
| 12. Work(ing) with your hands below knee level? | **0.66** |  |  | **0.77** |  | -0.48 |
| 21. Operating peddles with your feet? | **0.62** |  |  | **0.61** |  |  |
| 14. Moving heavy loads (more than 25kg)? | **0.62** |  |  | **0.74** | 0.34 | -0.47 |
| 23. Squatting often? | **0.61** |  |  | **0.72** |  | -0.55 |
| 13. Moving loads (more than 5kg)? | **0.57** |  |  | **0.75** | 0.39 | -0.57 |
| 17. Physical hard work? | **0.55** | 0.35 |  | **0.76** | **0.50** | -0.57 |
| 11. Work(ing) with your hands above shoulder level? | 0.45 | 0.32 | -0.39 | **0.67** | 0.46 | -0.57 |
| 5. Kneeling or squatting for long periods of time? | 0.42 |  | -0.36 | **0.62** | 0.34 | -0.56 |
| 20. Working with vibrating tools? | 0.38 |  |  | **0.53** |  | -0.44 |
| 10. Holding your wrist in a bent or twisted position for long periods of time? |  | **0.77** |  |  | **0.76** |  |
| 6. Making the same movement for long periods of time? |  | **0.77** | 0.39 |  | **0.74** |  |
| 8. Holding your neck in a bent forward or twisted position for long periods of time? |  | **0.74** |  |  | **0.72** |  |
| 9. Bending or twisting your neck often? |  | **0.73** |  |  | **0.74** |  |
| 18. Working in the same position for long periods of time? |  | **0.66** | **0.52** |  | **0.61** | **0.53** |
| 7. Working in a twisted posture for long periods of time? |  | **0.65** |  | 0.46 | **0.72** | -0.44 |
| 19. Working in uncomfortable postures? |  | **0.62** |  | 0.37 | **0.70** |  |
| 26. Doing repetitive tasks with arms, hands or fingers many times per minute? |  | 0.49 | 0.45 |  | **0.57** | 0.39 |
| 15. Exerting force with your arms or hands? | 0.42 | 0.47 |  | **0.66** | **0.61** | -0.53 |
| 16. Exerting maximal force? | 0.39 |  |  | **0.60** | **0.58** | -0.48 |
| 2. Sitting for long periods of time? |  |  | **0.87** | -0.40 |  | **0.88** |
| 3. VDU work for long periods of time? |  |  | **0.83** | -0.43 |  | **0.86** |
| 1. Standing for long periods of time? |  |  | -0.80 | 0.38 |  | -0.81 |
| 4. Walking long periods of time? | 0.40 |  | -0.47 | **0.61** |  | -0.64 |

Variance explained 38.9% 16.3% 5.9%

Total variance explained 61.2%

Factor loadings <0.3 are removed. Factor loadings >0.5 are given in bold

EFA: Exploratory Factor Analysis, VDU: Visual Display Unit
