# Supplementary material for: Assessing physical workload among people with musculoskeletal disorders: validity and reliability of the physical workload questionnaire
Source: BMC Musculoskelet Disord. 2022 Mar 24;23:282. doi: 10.1186/s12891-022-05222-y (PMC8944019; doi:10.1186/s12891-022-05222-y)
Supplement: Supplementary file 3 — Additional file 3. Table of Pattern and Structure matrix of two-factor solution after EFA with oblimin rotation. [file 12891_2022_5222_MOESM3_ESM.docx]

**Additional file 3.** Table of Pattern and Structure matrix of two-factor solution after EFA with oblimin rotation

| **Item** | **Pattern coefficient Structure coefficient Communalities** | | | | |
| --- | --- | --- | --- | --- | --- |
| Does your work involve… | **Factor 1** | **Factor 2** | **Factor 1** | **Factor 2** |  |
| 1. Standing for long periods of time? | **0.68** |  | **0.67** |  | 0.462 |
| 2. Sitting for long periods of time? | -0.72 | 0.36 | -0.68 |  | 0.589 |
| 3. VDU work for long periods of time? | -0.73 | 0.39 | -0.69 | 0.31 | 0.622 |
| 4. Walking long periods of time? | **0.74** |  | **0.72** |  | 0.541 |
| 5. Kneeling or squatting for long periods of time? | **0.71** |  | **0.72** |  | 0.527 |
| 6. Making the same movement for long periods of time? | -0.83 | **0.83** |  | **0.82** | 0.674 |
| 7. Working in a twisted posture for long periods of time? | **0.57** | **0.51** | **0.63** | **0.57** | 0.652 |
| 8. Holding your neck in a bent forward or twisted position for long periods of time? |  | **0.63** |  | **0.65** | 0.450 |
| 9. Bending or twisting your neck often? | 0.32 | **0.63** | 0.39 | **0.66** | 0.539 |
| 10. Holding your wrist in a bent or twisted position for long periods of time? |  | **0.74** |  | **0.75** | 0.571 |
| 11. Work(ing) with your hands above shoulder level? | **0.76** |  | **0.78** |  | 0.640 |
| 12. Work(ing) with your hands below knee level? | **0.77** |  | **0.78** |  | 0.605 |
| 13. Moving loads (more than 5kg)? | **0.81** |  | **0.82** |  | 0.685 |
| 14. Moving heavy loads (more than 25kg)? | **0.75** |  | **0.76** |  | 0.587 |
| 15. Exerting force with your arms or hands? | **0.74** | 0.36 | **0.78** | 0.44 | 0.736 |
| 16. Exerting maximal force? | **0.68** | 0.35 | **0.71** | 0.42 | 0.630 |
| 17. Physical hard work? | **0.82** |  | **0.84** | 0.32 | 0.761 |
| 18. Working in the same position for long periods of time? | -0.40 | **0.80** | -0.31 | **0.76** | 0.735 |
| 19. Working in uncomfortable postures? | 0.37 | **0.60** | 0.43 | **0.64** | 0.537 |
| 20. Working with vibrating tools | **0.59** |  | **0.59** |  | 0.356 |
| 21. Operating peddles with your feet? | **0.53** |  | **0.53** |  | **0.276** |
| 22. Climbing stairs? | 0.41 |  | 0.39 |  | **0.174** |
| 23. Squatting often? | **0.76** |  | **0.74** |  | 0.578 |
| 24. Walking on irregular surfaces? | **0.62** |  | **0.63** |  | 0.392 |
| 25. Sitting or moving on your knees? | **0.72** |  | **0.73** |  | 0.530 |
| 26. Doing repetitive tasks with arms, hands or fingers many times per minute? |  | **0.71** |  | **0.68** | 0.511 |

Eigenvalue 10.12 4.24

Variance explained before rotation 38.9% 16.3%

Total variance explained 55.2%

Factor loadings <0.3 are removed. Factor loadings >0.5 and communalities values <0.3 are given in bold

EFA: Exploratory Factor Analysis, VDU: Visual Display Unit
