# Supplementary material for: Assessing physical workload among people with musculoskeletal disorders: validity and reliability of the physical workload questionnaire
Source: BMC Musculoskelet Disord. 2022 Mar 24;23:282. doi: 10.1186/s12891-022-05222-y (PMC8944019; doi:10.1186/s12891-022-05222-y)
Supplement: Supplementary file 4 — Additional file 4 Table of missing data, floor- and ceiling effects of the PWQ subscales and items (n = 115). [file 12891_2022_5222_MOESM4_ESM.docx]

**Additional file 4.** Table of missing data, floor- and ceiling effects of the PWQ subscales and items (n=115)

| PWQ items. Does your work involve… | **Missing data,**  **n (%)** | **Lowest (%)** | **Highest (%)** |
| --- | --- | --- | --- |
| **Heavy physical work** | **8 (7.0)** | **9.3** | **0.0** |
| 1. Standing for long periods of time? | 1 (0.9) | 2.2 | 20.2 |
| 4. Walking long periods of time? | 1 (0.9) | 42.1 | 8.8 |
| 5. Kneeling or squatting for long periods of time? | 2 (1.7) | 64.6 | 3.5 |
| 7. Working in a twisted posture for long periods of time? | 1 (0.9) | 48.2 | 5.3 |
| 11. Work(ing) with your hands above shoulder level? | 1 (0.9) | 58.8 | 3.5 |
| 12. Work(ing) with your hands below knee level? | 1 (0.9) | 67.5 | 0.0 |
| 13. Moving loads (more than 5 kg)? | 1 (0.9) | 49.1 | 11.4 |
| 14. Moving heavy loads (more than 25 kg)? | 2 (1.7) | 63.7 | 0.9 |
| 15. Exerting force with your arms or hands? | 2 (1.7) | 42.5 | 14.2 |
| 16. Exerting maximal force? | 1 (0.9) | 59.6 | 7.9 |
| 17. Physical hard work? | 2 (1.7) | 59.3 | 6.2 |
| 20. Working with vibrating tools? | 1 (0.9) | 79.8 | 2.6 |
| 23. Squatting often? | 2 (1.7) | 52.2 | 6.2 |
| 24. Walking on irregular surfaces? | 1 (0.9) | 68.4 | 4.4 |
| 25. Sitting or moving on your knees? | 4 (3.5) | 72.2 | 1.8 |
| **Long lasting postures and repetitive movements** | **5 (4.3)** | **1.8** | **4.5** |
| 6. Making the same movement for long periods of time? | 1 (0.9) | 14.9 | 34.2 |
| 8. Holding your neck in a bent forward or twisted position for long periods of time? | 1 (0.9) | 30.7 | 13.2 |
| 9. Bending or twisting your neck often? | 2 (1.7) | 27.4 | 11.5 |
| 10. Holding your wrist in a bent or twisted position for long periods of time? | 2 (1.7) | 29.2 | 16.8 |
| 18. Working in the same position for long periods of time? | 1 (0.9) | 14.9 | 38.6 |
| 19. Working in uncomfortable postures? | 3 (2.6) | 32.1 | 8.9 |
| 26. Doing repetitive tasks with arms, hands or fingers many times per minute? | 1 (0.9) | 29.8 | 34.2 |
